# Factors Associated with Radiological Examination of Patients with Non-Specific Low Back Pain

**DOI:** 10.3390/jcm14207187

**Published:** 2025-10-12

**Authors:** Asma S. Alrushud, Muteb J. Alqarni, Salman Albeshan, Areej S. Aloufi, Mawaddah H. Aljohani, Mohammed A. Alqarni, Somyah A. Alhazmi, Yazeed I. Alashban, Dalia M. Alimam

**Affiliations:** 1 Department of Health Rehabilitation Sciences, College of Applied Medical Sciences, King Saud University, P.O. Box 10219, Riyadh 11433, Saudi Arabia; aalrashoud@ksu.edu.sa (A.S.A.);; 2 Physical Therapy Department, Royal Commission Medical Center, Yanbu 46451, Saudi Arabiaaljuhanimaw@rchsp.med.sa (M.H.A.); alqaranim@rchsp.med.sa (M.A.A.); 3 Department of Radiological Sciences, College of Applied Medical Sciences, King Saud University, P.O. Box 10219, Riyadh 11433, Saudi Arabiayalashban@ksu.edu.sa (Y.I.A.); 4 Medical Imaging Department, Royal Commission Medical Center, Yanbu 46451, Saudi Arabia

**Keywords:** low back pain, radiological imaging, physiotherapy, healthcare decision making

## Abstract

**Background/Objectives**: Non-specific low back pain (LBP), a highly prevalent musculoskeletal condition, may be associated with overuse of radiological imaging, despite clinical guidelines restricting its use to cases with suspected serious pathology. This study investigated demographic, clinical, and physiotherapy-related factors influencing radiological imaging use in patients with non-specific LBP. **Methods**: A retrospective cross-sectional study included 179 non-specific LBP patients from an outpatient physiotherapy clinic in Saudi Arabia. Patient data were anonymized and retrieved from electronic health records, including demographic, clinical, physiotherapy and imaging information. Independent variables included patient demographics, non-specific LBP characteristics, physiotherapy engagement, and pain-related outcomes. Descriptive, inferential, and multiple linear regression analyses were conducted to identify predictors of radiological imaging. **Results**: Among the total study sample (*n* = 179), 159 (88.8%) patients underwent radiological imaging, primarily X-ray (32.4%) and Magnetic Resonance Imaging (8.4%); 48.0% received multiple imaging modalities. Significant predictors of imaging use included gender (*p* < 0.001), higher body mass index (BMI) (*p* = 0.012), greater physiotherapist experience (*p* = 0.019), and presence of comorbidities (*p* = 0.023). Non-specific LBP medication use was negatively associated with imaging (*p* = 0.032). Physiotherapy engagement and pain-related outcomes showed no significant impact on imaging use. **Conclusions**: Gender, BMI, physiotherapist experience, and comorbidities could influence radiological imaging use in non-specific LBP patients. These findings highlight potential biases in imaging referral patterns and reinforce the need for adherence to evidence-based guidelines to prevent unnecessary imaging, reduce healthcare costs, and enhance patient care.

## 1. Introduction

Low back pain (LBP) is one of the most common musculoskeletal disorders globally and imposes a substantial public health burden. According to the 2021 Global Burden of Disease estimates, LBP remains the leading cause of years lived with disability (YLDs) worldwide, affecting over 600 million people in 2020 [1]. Although age-standardized prevalence and disability rates have marginally declined over the past three decades, the total number of people with LBP continues to rise, primarily driven by population growth and aging [1]. Despite its high burden, evidence-based clinical guidelines consistently recommend conservative management for non-specific LBP, reserving radiological imaging for cases where serious underlying pathology is suspected [2]. These guidelines emphasize that imaging should be guided by the presence of “red flag” symptoms such as suspicion of fracture, infection, malignancy, or neurological compromise. Nevertheless, in clinical practice, imaging is frequently used outside these recommendations, particularly in primary care and emergency settings [3].

Radiological procedures such as plain X-rays, magnetic resonance imaging (MRI), and computed tomography (CT) are often requested in patients with non-specific LBP, influenced by factors such as patient expectations, physician practice patterns, and the desire to exclude serious disease [2]. However, routine imaging in non-specific LBP rarely improves clinical outcomes and may lead to unnecessary interventions [4]. Overuse of imaging increases healthcare costs, exposes patients to potential harms, and may reinforce maladaptive illness beliefs, whereas underuse can delay the detection of serious pathology [5].

In a systematic review and meta-analysis, Jenkins et al. (2018) [6] reported a gap between guidelines and clinical practice, with around one-third of lumbar imaging referrals not aligning with recommended criteria. Inappropriate imaging occurred in nearly 28% of patients with symptoms < 6 weeks and in 9% with no red flags, while underuse was also evident, with more than half of patients with clear indications not imaged [6]. These findings underscore persistent problems of both overuse and underuse of imaging across healthcare systems.

Patient-related factors such as pain severity, chronicity, and healthcare beliefs, as well as clinician-related variables including decision-making styles and adherence to guidelines, can all influence imaging use [7,8]. Moreover, imaging may shape patients’ perceptions of their condition, either providing reassurance that supports conservative management or reinforcing beliefs about structural damage that undermine confidence in physiotherapy and other non-invasive strategies [9].

Regional data suggest further complexity. In Saudi Arabia, over 50% of participants with LBP believed that imaging was essential for optimal care, reflecting high expectations for diagnostic tests [10]. By contrast, a systematic review of international studies found that roughly one in four primary care patients and one in three emergency patients underwent imaging, far exceeding the <5% recommended by guidelines [11]. Such discrepancies highlight the role of cultural, clinical, and healthcare system factors in shaping imaging decisions [12,13].

Against this background, the present study examines demographic, clinical, and physiotherapy (PT)-related factors associated with the use of radiological examinations in patients with non-specific LBP. While much of the existing literature has focused on overall imaging rates and guideline adherence, less is known about the specific characteristics that drive imaging use in real-world practice. Importantly, physiotherapists are frequently the first point of contact for patients with LBP and play a central role in conservative management strategies. Their clinical decisions, communication styles, and level of adherence to evidence-based guidelines may substantially influence whether imaging is requested or deferred. However, the contribution of physiotherapy-related factors to imaging utilization remains underexplored, particularly within the Saudi healthcare context. By identifying demographic, clinical, and PT-related predictors of imaging use, this study aims to generate context-specific insights that can guide strategies to minimize unnecessary imaging and promote guideline-concordant care.

## 2. Materials and Methods

Clinical data from an outpatient physiotherapy clinic at the Royal Commission in Yanbu, Saudi Arabia, were used to perform a retrospective cross-sectional study. The clinic is government-funded, but operates under a hybrid model, offering both public and private services. Research ethics approval was received from the Royal Commission of Yanbu Institutional Review Board (RCYMC-EA-2023-01).

### 2.1. Study Population

Participants were identified by reviewing case files of patients with low back pain (LBP) at the clinic. Eligibility required individuals to be aged 18 years or older, have a physician-confirmed diagnosis, and have been referred for and received outpatient physiotherapy for non-specific LBP between 1 August 2021, and 31 March 2023. Exclusion criteria included: (1) indicators of serious pathology (e.g., malignancy, infection, cauda equina syndrome), (2) specific spinal conditions such as radiculopathy, (3) prior history of spinal surgery, or (4) pregnancy.

De-identified patient information was obtained from the clinic’s electronic health record system, where data are routinely documented by physiotherapists. In cases where multiple physiotherapists provided treatment, the “Physiotherapist of Record” was defined as the clinician who carried out the initial assessment. The anonymized dataset was exported into a secure Excel file containing all study variables.

### 2.2. Study Variables

The main outcome measure was the total number of radiological examinations undertaken during each “episode-of-care,” which was defined as a physician referral for low back pain. An episode of care commenced on the date of the patient’s initial physiotherapy assessment and concluded either on the documented discharge date or, if a discharge note was absent, on the last recorded visit without a subsequent appointment within 30 days [14]. Episodes without a discharge note beginning before 1 August 2021, or after 31 January 2023, were excluded to avoid partial episodes [15].

The selection of explanatory variables was guided by Dolot et al. [15] and the accessible dataset, with categorization based on Anderson and Newman’s Behavioral Model of Health Services Utilization [16]. The variables were organized into four categories: individual characteristics, individual need-related factors, clinical factors, and physiotherapy-related outcomes. Individual characteristics included age (in years), sex (male or female), body mass index (BMI, kg/m^2^), marital status (married, single, or not reported), and working status (relevant to the study population): (1) professionals (e.g., doctors, lawyers), (2) clerical/skilled labor, and (3) unemployed. Clinical-related factors included the duration of LBP in months, the occurrence of LBP (first episode or recurrence), the presence of comorbidities (e.g., hypertension, diabetes, asthma) based on self-reported or documented diagnosis, and use of LBP medication, including painkillers (yes or no). PT-related outcomes (the number of PT sessions per week, number of PT sessions per LBP episode, the time gap between the doctor’s referral and the first PT session (days)), pre-and post-treatment pain levels (0–10) as recorded in visual analog scale (VAS) [17], perceived functional improvement (assessed through the patient’s answer to the question “is there any improvement in your low back pain?” Yes or no) were also recorded. Compliance was defined as attendance at all scheduled physiotherapy sessions during the episode-of-care and was recorded as a binary variable (yes or no); the physiotherapist’s years of experience were rounded to the nearest whole year; any history of having PT sessions before LBP occurrence was recorded as “yes or no.” Data were presented for the total sample and according to the presence of having radiological examination (yes or no) to identify factors that influenced radiological examination for patients with LBP [18].

### 2.3. Statistical Analysis

Descriptive statistics, including mean, standard deviation (SD), median, interquartile range (IQR), and frequency distributions, were applied to summarize demographic, clinical, and physiotherapy-related characteristics of the participants. The distribution of continuous variables was examined for normality using the Shapiro–Wilk test together with visual assessment of histograms and Q-Q plots. As the continuous data were not normally distributed, group comparisons were carried out with Mann–Whitney U tests, while categorical data were analyzed using chi-square tests.

Correlations between the numbers of radiological procedures and the independent variables were examined using Spearman’s rho and point-biserial correlation analyses. The correlation coefficient (r) was classified as: very strong (≥0.7), strong (0.5–0.7), moderate (0.3–0.5), weak (0.1–0.3), and negligible (<0.1) [19].

Following the creation of dummy variables for categorical predictors, multiple linear regression using the standard enter approach was applied to evaluate the relationship between the number of radiological procedures and the independent variables. The results were expressed as β coefficients along with adjusted R^2^ values [20]. Cases with missing outcome data were kept in the analysis, as their exclusion could introduce bias by substantially reducing the sample size and limiting its representativeness, which in turn might weaken statistical power and affect the generalizability of the findings.

For linear regression analyses, all potential predictors (demographic, clinical, and PT-related factors) were included in models. Statistical significance was defined at *p* < 0.05, and all data analyses were carried out with SPSS Statistics version 28 (IBM Corp., New York, NY, USA); where applicable, results are presented with 95% confidence intervals.

## 3. Results

Demographic characteristics of 179 LBP patients were analyzed, of which 159 (88.6%) underwent radiological examination and 20 did not. Patients’ mean age was 43.51 years (SD = 13.60), with no significant difference between those who did and those who did not undergo radiological examination (*p* = 0.130). BMI was comparable across these two groups (*p* = 0.258). Gender distribution differed significantly (*p* = 0.036), with proportionally more females undergoing radiological examinations. Neither marital nor working status was significantly associated with imaging use (Table 1).

As shown in Table 2, clinical characteristics were similar between patients with and without radiological examinations. Duration of LBP (*p* = 0.347), recurrence of episodes (*p* = 0.834), and medication use (*p* = 0.089) were not significantly associated with imaging. A possible association was observed with having comorbidities (*p* = 0.059), suggesting a trend toward increased imaging among patients with comorbidities.

X-ray was the most common single imaging modality (58 patients, 32.4%). MRI alone was used on 15 patients (8.4%). Many patients (86, 48.0%) underwent multiple imaging examinations, including combinations of X-ray, MRI, and/or CT scans.

The assessment of PT-related factors between patients who underwent radiological examination revealed no significant differences across all factors (Table 3). The number of PT sessions per week (*p* = 0.184) and per LBP episode (*p* = 0.372) did not differ significantly between groups. Similarly, the gap in time between referral and PT initiation was comparable (*p* = 0.786). Pain scores before and after PT were similar between groups (*p* = 0.942 pre-PT, *p* = 0.905 post-PT). Perceived functional improvement (*p* = 0.542) and compliance with PT sessions (*p* = 0.940) were not significantly associated with undergoing imaging.

Correlation analysis using Spearman’s rho examined relationships between the numbers of radiological procedures performed and various demographic, clinical, and PT-related factors (Table 4). Significant positive correlations were found with BMI (r = 0.240, *p* = 0.002), physiotherapist experience (r = 0.245, *p* < 0.001), gender (r = 0.349, *p* < 0.001), and comorbidities (r = 0.173, *p* = 0.023). These results indicate that higher BMI, female gender, greater physiotherapist experience, and the presence of comorbidities are linked to a greater likelihood of receiving radiological examinations. The use of LBP medications was negatively correlated (r = −0.161, *p* = 0.032), indicating that patients taking medication for LBP were less likely to undergo imaging. Other factors (age, LBP duration, referral gap, PT session frequency, pain scores, and perceived functional improvement) showed no statistically significant correlations with the number of radiological procedures.

To explore the relationship between radiological procedure counts and the different independent factors, multiple linear regression analyses were conducted (Table 5 and Table 6). For binary variables and numbers of radiological exams, the overall model was statistically significant, F (8, 165) = 3.659, *p* < 0.001, indicating that the predictors collectively explained a significant proportion of the variance in the dependent variable (Table 5).

Among predictors, gender (β = 1.232, SE = 0.332, t = 3.715, *p* < 0.001) was significantly associated with the number of radiological procedures, with females undergoing more exams than males. Other variables (marital status, LBP recurrence, medication use, presence of medical conditions, previous PT sessions, perceived functional improvement, and compliance with PT) were not significantly (*p* > 0.05) associated with the number of radiological procedures.

Multiple linear regression was used to assess the associations between several continuous predictors and the number of radiological examinations (Table 6). The predictors included in the model were age, body mass index (BMI), duration of LBP, interval between referral and physiotherapy, pain scores before and after PT, physiotherapist experience, weekly frequency of PT sessions, and the total number of PT sessions per episode.

Among predictors, BMI (β = 0.057, *p* = 0.012) and physiotherapist years of experience (β = 0.137, *p* = 0.019) were statistically significant, suggesting that a higher BMI and greater physiotherapist experience were associated with an increased number of radiological exams. All other predictors (age, LBP duration, time gap, pre- and post-PT pain scores, and the number of PT sessions) were not significant (*p* > 0.05); none has a strong independent effect on the number of radiological exams in this model.

## 4. Discussion

This study aimed to explore determinants of the number of radiological procedures undertaken per episode-of-care in patients with LBP. The final model highlighted several significant predictors (gender, BMI, and physiotherapist years of experience) that most correlate with the likelihood of undergoing radiological examinations.

Among the 179 LBP patients, it was found that 159 (88.8%) underwent radiological imaging. In contrast, a meta-analysis encompassing eight primary care studies reported that only 24.8% of adults presenting with low back pain underwent imaging, including medical, chiropractic, physiotherapy, and osteopathy settings [21]. Similarly, studies evaluating imaging practices in older adults receiving medical care for low back pain have reported rates ranging from 23.7% [22] to 38.3% [23] within the first four weeks of presentation. This discrepancy may reflect differences in healthcare settings, patient populations, or clinical practices between regions. In the current study, this percentage (88.8%) indicates a potential overuse of radiological procedures, even in cases of non-specific LBP, where imaging is not routinely recommended. For this condition, clinical practice guidelines advise against unnecessary use of radiological imaging, unless red flags or specific indications are present [21].

Female patients underwent significantly more imaging procedures than males, consistent with previous findings that suggested gender differences in healthcare use and pain management [24,25,26]. Gender may influence the decision-making process or diagnostic procedures in LBP because of differences in clinical needs, healthcare provider practices, or patient expectations. Differences have been consistently demonstrated between genders in pain perception, description, coping strategies, and treatment benefits [27,28,29]. The biopsychosocial model of LBP emphasizes the interplay between biological, psychological, and social factors in the experience of pain, and that women may have a stronger need for reassurance regarding the nature of their condition than men [30]. Given that women with chronic pain conditions frequently report greater pain intensity scores, shorter pain tolerance, and engage in catastrophizing behavior, this can lead to increased healthcare use and a greater likelihood of their undergoing imaging [27,28].

Pain is inherently subjective, and pain scales commonly used in research and clinical practice assess pain reports that can be influenced by social factors such as gender. These gender norms shape pain perception and responses, which can introduce bias in healthcare decision-making [29]. Another potential bias in healthcare decision-making could be related to patients with higher BMI. Healthcare providers may perceive higher BMI to be a risk and request more radiological procedures to rule out serious spinal pathology, indicating that patient characteristics and provider expertise play a role in imaging decisions [27,28,29]. Patients with a higher BMI may also experience more severe or persistent symptoms, leading physicians to request imaging to rule out structural abnormalities [31]. Obesity may be associated with increased mechanical stress on the spine, which can contribute to conditions such as degenerative disc disease and osteoarthritis, potentially justifying the use of imaging in some cases [31,32].

Healthcare providers in our study may have been more likely to order additional diagnostic tests to provide reassurance, even if clinical indications did not necessitate further imaging. Furthermore, more experienced physiotherapists should rely more on clinical assessment rather than imaging. However, patient expectations or characteristics may influence their decisions, even if clinical practice guidelines advise against routine imaging. Clinicians are more likely to recommend psychological treatment for women and analgesics for men, even when pain intensity is equal [29,32]. This socialization could explain the findings of increased radiological procedures in women because their pain expressions may be taken more seriously or more readily investigated than men, who may be seen as stoic and less likely to verbalize pain [25,33].

Our results showed no significant association between physiotherapist-related factors and whether patients underwent radiological examinations. Nevertheless, the overall imaging rate in our study was high (88.8%), suggesting that factors beyond PT influence likely contribute to imaging decisions. Previous research has identified several potential explanations. Patient expectations often drive imaging requests, with many individuals perceiving imaging as necessary to validate their pain or ensure appropriate care [10]. Clinician beliefs, including a desire to avoid missed diagnoses, may also contribute to higher imaging rates [2]. Furthermore, international studies have reported that a substantial proportion of patients undergoing low back imaging did not meet guideline-based criteria, with inappropriate imaging observed even when provider-related factors were not significant, highlighting a persistent gap between clinical practice and guideline recommendations [6]. Although physiotherapists may not directly refer patients for imaging, they play a crucial role in guiding care, educating patients, and coordinating with physicians to support evidence-based imaging decisions and minimizing unnecessary procedures.

Our findings indicate that advanced imaging techniques, particularly MRI and combined imaging approaches, are associated with a higher number of radiological procedures. X-ray was the most common single imaging modality, performed on 58 patients (32.4%), while MRI alone was used on 15 patients (8.4%). A large proportion of patients (48.0%) underwent multiple imaging examinations, including combinations of X-ray, MRI, and/or CT scans. More complex imaging techniques generally provide greater detail about underlying conditions, potentially necessitating additional imaging to fully understand the scope of the issue [34]. However, it is important to ensure that imaging is clinically justified and not overly driven by a perceived need for exhaustive reassurance or physician preference. Unnecessary procedures contribute to increased healthcare costs and patient anxiety [33], and imaging for non-specific LBP is widely recognized as one of the most common low-value diagnostic practices [35]. Evidence from healthcare provider practices also demonstrates an increase in imaging rates for patients with new LBP in general practice, despite guidelines advising against routine imaging [36,37]. Using resources wisely in such cases can help reduce unnecessary costs, improve patient outcomes, and support the principles of value-based care by focusing on effective, evidence-based treatments [38].

We acknowledge several study limitations. (1) The cross-sectional design restricts the capacity to draw causal inferences between the identified factors and the number of radiological procedures, allowing only the detection of associations. (2) Conducting the study within a single healthcare setting may restrict the generalizability of the results to other populations and healthcare systems. (3) Although demographic, clinical, and PT-related factors were examined, other potential influences, such as healthcare provider decision-making biases, patient anxiety levels, and socioeconomic status, were not assessed. (4) The reliance on retrospective data may have introduced recall or documentation biases in the recorded clinical and imaging information. Future longitudinal studies could provide better insights into causal relationships with larger and multicenter samples. Finally, (5) we did not assess the potential impact of physiotherapist gender or gender-matching with patients on clinical decision-making. While preliminary observations suggest a predominance of female providers, we cannot rule out the possibility that gender-related factors such as perceived empathy or patient comfort may have influenced imaging referral patterns. Future studies should consider collecting data on the dynamics of physiotherapist–patient gender interactions to better understand their potential impact on care decisions.

Prospective clinical implications based on this study’s findings include healthcare provider prioritization of evidence-based guidelines to avoid unnecessary imaging to reduce costs, patient anxiety, and potential overdiagnosis. Healthcare providers should also be aware of potential biases in decision-making related to patient characteristics (e.g., gender). Diagnostic and treatment decisions for LBP must be based on clinical evidence and an individual’s needs rather than gender-related assumptions. It should also be noted that the clinic in this study operates under a hybrid model, offering both public and private services. While cost considerations may differ between these two sectors, reducing unnecessary imaging remains relevant in both contexts to optimize resource use in the public sector and to minimize patient burden and potential overdiagnosis in the private sector. Additionally, although physiotherapists do not always directly refer patients for imaging—since referral practices depend on healthcare policies—they play a key role in guiding patient care, providing advice and education, and coordinating with physicians to support appropriate use of imaging.

## 5. Conclusions

Associations were observed between gender, BMI, physiotherapist experience, and the number of radiological procedures per episode of care among LBP patients. Female patients appeared more likely to undergo imaging, which may reflect potential biases in clinical decision-making or psychosocial factors influencing pain experiences, though these explanations require further investigation. Similarly, the observed associations with higher BMI and greater physiotherapist experience suggest that both patient- and provider-related characteristics may influence imaging use.

Given the cross-sectional nature of this study and the relatively weak associations observed, these findings should be interpreted with caution. The results do not establish causality but instead highlight possible explanations that warrant testing in future prospective studies.

Efforts to optimize imaging for LBP should continue to emphasize adherence to clinical guidelines and evidence-based practices to prevent unnecessary procedures, reduce costs, and minimize potential harm. Further research, particularly using longitudinal designs and exploring psychosocial and system-level factors, is needed to better understand the drivers of imaging use and to develop strategies that support guideline-concordant, value-based care.

## Figures and Tables

**Table 1:** Sample demographic characteristics.

Characteristic	Total Sample (*n* = 179)	Had Radiological Tests (*n* = 159)	No Radiological Test (*n* = 20)	*p* Value *
Age				
Mean (SD)	43.51 (13.60)	43.12 (13.4)	48.55 (13.57)	
Median (IQR)	42 (23)	41 (23)	50.5 (21)	
Minimum–Maximum	18–70	18–70	25–70	0.130
BMI				
Mean (SD)	28.87 (6.44)	28.7 (6.58)	29.89 (3.33)	
Median (IQR)	28.67 (8.40)	28.3 (8.6)	30.4 (3.57)	
Minimum–Maximum	14.60–53.01	14.6–53.01	23.46–35.65	0.258
Gender *n* (%)				
Male	39 (21.8)	31 (19.5)	8 (40.0)	
Female	140 (78.2)	128 (80.5)	12 (60.0)	**0.036**
Marital Status *n* (%)				
Married	136 (76.0)	121 (76.1)	15 (75.0)	
Single	39 (21.8)	34 (21.4)	5 (25.0)	
Unknown	4 (2.2)	4 (2.5)	0	0.736
Working Status *n* (%)				
Professionals	16 (8.9)	15 (9.4)	1 (5.0)	
Clerical and skilled labor	8 (4.5)	6 (3.8)	2 (10.0)	
Unemployed	155 (86.6)	138 (86.8)	17 (85.0)	0.379

* Chi-square for categorical and binary variables, and Mann–Whitney U test for continuous variables. Values in bold *p* indicate statistical significance at *p* < 0.05. SD Standard Deviation, IQR Interquartile range, BMI Body Mass Index.

**Table 2:** Clinical characteristics of LBP patients who did and did not receive radiological tests.

	Total Sample (*n* = 179)	Radiological Test (*n* = 159)	No Radiological Test (*n* = 20)	*p*-Value *
LBP duration (in months)				
Mean (SD)	11.81 (16.12)	11.68 (16.04)	12.84 (17.19)	
Median (IQR)	6 (10.0)	6 (10.0)	4 (10.0)	
Minimum–Maximum	0.1–120	0.1–120	0.50–60	0.347
Occurrence of LBP *n* (%)				
1st episode	159 (88.8)	141 (88.7)	18 (90.0)	
Recurrence	20 (11.2)	18 (11.3)	2 (10.0)	0.834
Taking LBP medication *n* (%)				
Yes	135 (75.4)	123 (77.4)	12 (60.0)	
No	44 (24.6)	36 (22.6)	8 (40.0)	0.089
Presence of comorbidities *n* (%)				
Yes	47 (26.3)	40 (25.2)	7 (35.0)	
No	127 (70.9)	116 (73.0)	11 (55.0)	
Unknown	5 (2.8)	3 (1.8)	2 (10.0)	0.059

* Chi-square for categorical and binary variables and Mann–Whitney U for continuous variables.

**Table 3:** Comparison of physiotherapy characteristics of LBP patients with and without radiological tests.

	Total Sample (*n* = 179)	Radiological Test (*n* = 159)	No Radiological Test (*n* = 20)	*p*-Value *
Number of PT sessions per week				
Mean (SD)	1.54 (0.54)	1.52 (0.53)	1.70 (0.57)	
Median (IQR)	2 (1)	2 (1)	2 (1)	
Minimum–Maximum	1–3	1–3	1–3	0.184
Number of PT sessions per LBP episode				
Mean (SD)	5.19 (2.59)	5.14 (2.19)	5.55 (4.80)	
Median (IQR)	5 (2)	5 (2)	4 (3)	
Minimum–Maximum	2–23	2–13	2–23	0.372
Time gap between referral and PT (days)				
Mean (SD)	5.83 (1.35)	5.83 (1.37)	5.85 (1.22)	
Median (IQR)	6 (2)	6 (2)	6 (1)	
Minimum–Maximum	2–10	2–10	3–8	0.942
Pre-PT pain score				
Mean (SD)	2.06 (1.61)	2.06 (1.61)	2.00 (1.62)	
Median (IQR)	2 (2)	2 (2)	2 (2)	
Minimum–Maximum	0–7	0–7	0–6	0.905
Physiotherapist experience (years)				
Mean (SD)	4.82 (2.35)	4.89 (2.34)	4.25 (2.40)	
Median (IQR)	4 (3)	4 (3)	4 (3)	
Minimum–Maximum	2–12	2–12	2–12	0.181
Perceived functional improvement *n* (%)				
Yes	26 (14.5)	24 (15.1)	2 (10.0)	
No	153 (85.5)	135 (84.9)	18 (90.0)	0.542
Compliance with PT session *n* (%)				
Yes	106 (59.2)	94 (59.1)	12 (60.0)	
No	73 (40.8)	65 (40.9)	8 (40.0)	0.940
History of PT before LBP *n* (%)				
Yes	156 (87.2)	138 (86.8)	18 (90.0)	
No	23 (12.8)	21 (13.2)	2 (10.0)	0.686

* Chi-square for categorical and binary variables and Mann–Whitney U for continuous variables. Physiotherapy (PT), low back pain (LBP), standard deviation (SD), inter-quartile range (IQR).

**Table 4:** Correlations between the number of performed radiological procedures and predictors.

Variable	Characteristic	Number of Performed Radiological Procedures
	*n*	179
Age	Correlation	0.890
	*p* value	0.234
	*n*	166
Body Mass Index (BMI)	Correlation	0.240
	*p* value	**0.002**
	*n*	171
Duration of LBP	Correlation	0.142
	*p* value	0.064
	*n*	179
Gap between referral and 1st session	Correlation	−0.012
	*p* value	0.872
	*n*	179
Number of PT sessions per week	Correlation	−0.103
	*p* value	0.169
	*n*	179
Number of PT sessions per episode	Correlation	0.089
	*p* value	0.237
	*n*	179
Pre-pain VAS	Correlation	0.078
	*p* value	0.300
	*n*	179
Post pain VAS	Correlation	0.073
	*p* value	0.331
	*n*	179
Physiotherapist’s years of experience	Correlation	0.245
	*p* value	**<0.001**
	*n*	179
Gender	Correlation	0.349
	*p* value	**<0.001**
	*n*	179
Occurrence of LBP	Correlation	0.113
	*p* value	0.132
	*n*	179
Medications for LBP	Correlation	−0.161
	*p* value	**0.032**
	*n*	174
Presence of comorbidities	Correlation	0.173
	*p* value	**0.023**
	*n*	179
Had previous PT	Correlation	−0.145
	*p* value	0.052
	*n*	179
Perceived functional improvement	Correlation	0.008
	*p* value	0.917
	*n*	179
Complete PT session	Correlation	0.048
	*p* value	0.521

Values in bold indicate statistical significance at *p* < 0.05.

**Table 5:** Comparison of physiotherapy characteristics of low back pain patients with and without radiological tests.

Predictor Variable	β Coefficient ^1^	SE	t-Value	*p* Value
Gender (female vs. male)	1.232	0.332	3.715	**<0.001**
Marital status (married vs. not married)	0.400	0.286	1.402	0.163
Occurrence (recurrence vs. 1st episode)	0.236	0.528	0.446	0.656
Medication for LBP (taking medication vs. not)	−0.267	0.289	−0.924	0.357
Presence of comorbidities (yes vs. no)	0.398	0.280	1.421	0.157
Having previous PT sessions (yes vs. no)	−0.526	0.495	−1.063	0.289
Perceived functional improvement (yes vs. no)	0.000	0.350	−0.001	0.999
Compliance with PT session (yes vs. no)	−0.004	0.247	−0.017	0.987

^1^ The multiple linear regression was adjusted for all variables; values in bold indicate statistical significance at *p* < 0.05. Low back pain (LBP), physiotherapy (PT), standard error (SE).

**Table 6:** Multiple linear regression predicting the number of radiological exams (continuous factors).

Predictor Variable	β Coefficient ^1^	SE	t-Value	*p* Value
Age (years)	0.003	0.011	0.315	0.753
BMI (kg m^−2^)	0.057	0.022	2.544	**0.012**
LBP duration (months)	0.003	0.008	0.356	0.722
Time gap between referral and PT (days)	0.002	0.012	0.165	0.869
Pre-PT pain score (VAS)	0.009	0.106	0.084	0.933
Post-PT pain score (VAS)	0.112	0.095	1.177	0.241
Physiotherapist’s years of experience	0.137	0.058	2.375	**0.019**
Number of PT sessions per week	−0.189	0.258	−0.732	0.465
Number of PT sessions per episode	0.016	0.056	0.279	0.780

^1^ The multiple linear regression was adjusted for all variables; values in bold indicate statistical significance at *p* < 0.05. Physiotherapy (PT), visual analog scale (VAS), standard error (SE).

## Data Availability

The raw data file is available from the corresponding author upon reasonable request.

## Abbreviations

The following abbreviations are used in this manuscript:

LBP	low back pain
BMI	body mass index
MRI	Magnetic Resonance Imaging
CT	Computed Tomography
PT	physiotherapy
SD	standard deviation
IQR	interquartile range
